# Visual motion sensitivity in descending neurons in the hoverfly

**DOI:** 10.1007/s00359-020-01402-0

**Published:** 2020-01-28

**Authors:** Sarah Nicholas, Richard Leibbrandt, Karin Nordström

**Affiliations:** 1 Centre for Neuroscience, Flinders University, GPO Box 2100, Adelaide, SA 5001 Australia; 2 Department of Neuroscience, Uppsala University, Box 593, 751 24 Uppsala, Sweden

**Keywords:** Looming, Target detection, Optic flow, Ventral nerve cord, Insect vision

## Abstract

Many animals use motion vision information to control dynamic behaviors. For example, flying insects must decide whether to pursue a prey or not, to avoid a predator, to maintain their current flight trajectory, or to land. The neural mechanisms underlying the computation of visual motion have been particularly well investigated in the fly optic lobes. However, the descending neurons, which connect the optic lobes with the motor command centers of the ventral nerve cord, remain less studied. To address this deficiency, we describe motion vision sensitive descending neurons in the hoverfly *Eristalis tenax*. We describe how the neurons can be identified based on their receptive field properties, and how they respond to moving targets, looming stimuli and to widefield optic flow. We discuss their similarities with previously published visual neurons, in the optic lobes and ventral nerve cord, and suggest that they can be classified as target-selective, looming sensitive and optic flow sensitive, based on these similarities. Our results highlight the importance of using several visual stimuli as the neurons can rarely be identified based on only one response characteristic. In addition, they provide an understanding of the neurophysiology of visual neurons that are likely to affect behavior.

## Introduction

Many animals rely on visual information for their survival. In particular, motion vision provides crucial information when navigating through the surroundings, when searching for prey, avoiding predators, defending a territory, and for a myriad of other behaviors. Motion vision has been particularly well investigated in insects, as they are amenable to both electrophysiological investigation and behavioral quantification (see, e.g. Mauss et al. 2017). Furthermore, most flying insects are highly specialized visual animals. Indeed, even in nocturnal insects a large proportion of the brain volume is dedicated to visual processing (Stöckl et al. 2016).

Many insects have an anterior, cephalic brain which is largely devoted to processing sensory information, and more posterior ganglia in the thorax and abdomen, which generate the motor commands that control, e.g. neck, wing and leg movements. In flies, three paired thoracic ganglia are fused with the abdominal ganglion to form the ventral nerve cord (Power 1948), which is connected to the anterior brain via the cervical connective containing both ascending and descending neurons. Ascending neurons provide sensory and motor feedback to the brain, whereas descending neurons carry sensory and motor-related information from the brain to central pattern generators in the posterior ganglia. Descending neurons can thus initiate and modify behavior based on sensory input, and other higher order processing that takes place in the brain. Importantly, whereas the anterior brain of an adult *Drosophila* contains about 100,000 neurons (Zheng et al. 2018) there are only 1100 descending neurons (Hsu and Bhandawat 2016). The descending neurons thus constitute a bottleneck for information conveyed to motor command centers.

In fly motion vision most of the attention has been given to the neurons in the optic lobes (for review, see, e.g. Borst 2014). Importantly, however, the responses of descending neurons are not always directly deducible from the responses of their presumed pre-synaptic counterparts (Wu et al. 2016; Chen et al. 2018). For example, in constrained animals the responses of descending optic flow sensitive neurons fluctuate less when stimulated with naturalistic scenery than the pre-synaptic lobula plate tangential cells (LPTCs, Wertz et al. 2009a). In addition, whereas lobula small target motion detectors (STMDs) respond robustly to target motion against a moving background (Nordström et al. 2006), target-selective descending neurons (TSDNs) do not (Nicholas et al. 2018a). As the descending neurons provide input to motor command centers in the thoracic ganglia (Hsu and Bhandawat 2016; Namiki et al. 2018), it is important to understand their response properties. Indeed, orchestrating complex behavior using only 1100 descending neurons (Hsu and Bhandawat 2016), requires efficient integration of sensory input and motor output, as well as higher-order processing, such as learning.

Descending neurons synapse with motor neurons or with interneurons that themselves connect with motor neurons (Venkatasubramanian and Mann 2019). Some descending neurons have been described as command neurons, i.e. they are necessary and sufficient for the generation of a specific behavior (Kupfermann and Weiss 2001). A classic example of this is the giant fiber, which generates escape responses to rapidly approaching stimuli (for review, see Card 2012). However, more recent work has shown that giant fiber activity is not always correlated with an escape jump (Fotowat et al. 2009; von Reyn et al. 2014). Several recent papers instead suggest that combined activity of groups of descending neurons determine behavioral output (Gonzalez-Bellido et al. 2013; Namiki et al. 2018), or even that different descending neurons generate the same behavioral output (Cande et al. 2018; Ache et al. 2019a). In fact, the same descending neuron can control several distinct behaviors depending on its spike rate (McKellar et al. 2019). In addition, many descending neurons are inhibitory (Hsu and Bhandawat 2016) suggesting that neuronal activity does not necessarily imply activation of a behavior.

Here we describe descending neurons sensitive to visual motion in the hoverfly *Eristalis tenax*. Hoverflies are named after their ability to hover, nearly stationary in mid-air, using visual cues. Male hoverflies guard their territory from intruding conspecifics which they pursue at high speed (Wellington and Fitzpatrick 1981). Such target pursuit is believed to be based on sharply tuned STMD neurons in the lobula (Collett and King 1975; O’Carroll 1993; Nordström et al. 2006), which likely provide input to TSDNs, as they show similar size tuning (Nicholas et al. 2018a). If the hoverfly is instead on collision course with a conspecific, or if a predator is rapidly approaching, the stimulus changes from a small drifting target to a rapidly expanding, looming object. The neural encoding of looming stimuli is well studied in locusts and more recently in *Drosophila* (for review, see Card 2012), but has not been investigated in hoverflies.

During target pursuit, the hoverfly’s own motion through the world generates optic flow, which is processed by well-studied LPTCs (Mauss et al. 2017). There are about 60 different LPTCs in the blowfly brain, working as matched filters to different types of optic flow (Franz and Krapp 2000), with similar neurons described in hoverflies (Buschbeck and Strausfeld 1997; Nordström et al. 2008). In blowflies and *Drosophila*, the output of LPTCs is spatially pooled in a handful of neurons in the descending nerve cord (Wertz et al. 2008, 2009b; Suver et al. 2016). These might be similar to the hoverfly widefield sensitive descending neurons (Nicholas et al. 2018a), whose receptive fields have not been described previously.

We refer to the descending neurons described here as looming sensitive, optic flow sensitive or target selective. TSDNs are identified by their sharp size tuning, and lack of response to widefield motion (Nicholas et al. 2018a). We show that the looming sensitive and optic flow sensitive neurons can be identified based on the receptive fields as mapped with sinusoidal gratings. Based on their receptive fields, the optic flow sensitive neurons are similar to previously described neurons in blowflies and *Drosophila* (Wertz et al. 2008, 2009b; Suver et al. 2016). We show that the looming sensitive descending neurons respond to looming stimuli before the simulated time of collision, like their *Drosophila* and locust counterparts (Fotowat et al. 2011; von Reyn et al. 2014). Our results are important as they provide an understanding of the response properties of visual motion-sensitive descending neurons, which are likely to have a more direct effect on behavior than the more extensively studied LPTCs.

## Materials and methods

### Animals and electrophysiology

*Eristalis tenax* hoverflies were reared and housed as described previously (Nicholas et al. 2018b). We recorded from 118 neurons in 100 male hoverflies. At experimental time, the animal was immobilized ventral side up with a beeswax and resin mixture, and a small hole was cut over the cervical connective at the anterior end of the thorax. A sharp polyimide-insulated tungsten electrode (2 MOhm, Microprobes, Gaithersburg, USA) was inserted into the cervical connective, with mechanical support given by a small wire hook. The animal was grounded via a silver wire inserted into the ventral cavity, which also served as the recording reference.

Extracellular signals were amplified at 1000 × gain and filtered through a 10- to 3000-Hz bandwidth filter on a DAM50 differential amplifier (World Precision Instruments), with 50 Hz noise removed with a HumBug (Quest Scientific, North Vancouver, Canada). The data were digitized via a Powerlab 4/30 (ADInstruments, Sydney, Australia) and acquired at 40 kHz with LabChart 7 Pro software (ADInstruments).

### Visual stimuli

Visual stimuli were displayed on an Asus LCD screen (Asus, Taipei, Taiwan), using custom written software based on the Psychophysics toolbox (Brainard 1997; Pelli 1997) in Matlab (Mathworks). The screen had a refresh rate of 165 Hz, a spatial resolution of 2560 × 1440 pixels, and linearized contrast with a mean illuminance of 200 Lux. *Eristalis* males were placed ventral side up, perpendicular to the screen, at a distance of 6.5 cm. They were facing the middle of screen, giving a projected screen size of 155° × 138°.

All targets were displayed as 15 × 15 pixel squares, moving at a velocity of 900 pixels per second, unless otherwise indicated. When converted to angular values, and taking a typical, frontal receptive field size into account (Fig. 2c), this corresponds to a 3° × 3° target, moving at 130 °/s. For size tuning a bar moved in each neuron’s preferred direction. The width, or the side parallel to the direction of travel, was fixed at 3°, and the height, or the side perpendicular to the direction of travel, was varied between 0.2° and 138° (the extent of the screen). The center of each bar was aligned with each neuron’s area of peak sensitivity to target motion. There was a minimum 3.5 s interval between the presentation of each bar, which was determined to be enough to avoid habituation between presentations (data not shown).

Sinusoidal gratings were shown as full-screen, full contrast stimuli, with an average wavelength of 7° (0.14 cycles/°) and drifting at a temporal frequency of 5 Hz, unless otherwise indicated. For receptive field mapping we used a modified version of previously described methods (Straw et al. 2006), where a local sinusoidal grating stimulus (38° × 38°) moved in a series of eight different directions presented in a pseudorandom order for 0.36 s each. The stimuli were placed in an overlapping tiling fashion so that the 48 squares covered the majority of the screen. There was a minimum 1 s interval between each stimulation, with the 800 ms immediately preceding each stimulation used to calculate spontaneous spiking activity.

To generate looming stimuli we used previously described methods (Fotowat and Gabbiani 2007) where the circular stimulus had an *l*/|*v*| of 10 ms, where *l* refers to the half-width of the stimulus (16 cm), and *v* to the approach velocity (16 m/s). The *l*/|*v*| has been shown to be the important parameter for looming stimuli, and the same value can be generated by smaller stimuli moving slower, or larger stimuli moving faster (Gabbiani et al. 1999). The black circular stimulus was displayed on a white screen where it expanded from 1° to a final diameter of 117° over 1 s. It then remained in full size on the screen for 1 s. We used two controls: The first was a luminance-matched, motion-free control with a diameter of 117°, which changed from white to black over 1 s to match the luminance change over time associated with the looming stimulus. It then remained on the screen for 1 s. The second control was an appearance control where a black disc with diameter of 117° appeared and remained on the screen for 1 s. There was a minimum 60 s between each looming stimulus presentation.

3-dimensional “starfield” stimuli were generated by using the “Screen(DrawDots)” function in the Psychophysics toolbox (Brainard 1997; Pelli 1997). The dots were simulated as a cloud of 2 cm spheres with a density of 100 per m^3^, generating 6400 dots randomly positioned within a 4-m cube with the hoverfly placed in its center. We displayed the approximately 1200 dots appearing anterior to the fly by 3-dimensional projection of the cloud onto the screen. Dots that were simulated to be closer than 6 cm or further away than 200 cm from the fly (along an axis perpendicular to the screen) were not displayed. Dots that were more distant from the fly were displayed smaller on-screen, and were also brighter, with luminance linearly interpolated from black to white across the visible region of space. We simulated six types of optic flow, three translations (sideslip, lift and thrust) and three rotations (pitch, yaw, roll), using the hoverfly’s midline and equator for alignment. For translations, all dots in the 3-dimensional space were translated at a velocity of 50 cm/s, and for rotations, all dots were rotated at 50 °/s. For translations, the subsequent two-dimensional projection meant that projected circles corresponding to dots that were nearer to the fly moved faster across the screen.

### Data analysis and statistics

Spike sorting of extracellular data was done using LabChart 7 Pro with the Spike Histogram Add-On (ADInstruments, Sydney, Australia), which uses the action potential amplitude and width to identify responses from individual neurons. In addition, we quantified the interspike intervals from the resulting spike trains, with the absence of any interspike intervals shorter than 1 ms used as a cut-off criterion. All further data analysis was done in Matlab.

For receptive field mapping we modified previously described techniques to calculate the local preferred direction and local motion sensitivity (Krapp and Hengstenberg 1997; Nordström et al. 2006; Straw et al. 2006). We quantified the mean response for each direction after removing the first 100 ms of the response to avoid any initial onset transients (Nordström and O’Carroll 2009b; Nordström et al. 2011). For each spatial location we fitted a cosine function to the response to each direction of motion, and extracted preferred direction and the amplitude of the function. In addition, we calculated the average spiking frequency for each location, and after subtracting the spontaneous rate, calculated for 800 ms preceding stimulation, we spatially interpolated this 10 times. As the animal was ventral side up during recordings, we rotated the resulting receptive fields to display them dorsal side up. Most neurons (70 out of 118) had a receptive field in the left visual field. The data from neurons that had a receptive field in the right visual field were flipped along the midline, assuming that the left and right visual fields were mirror images of each other. From each resulting receptive field we calculated the area and its center using the *contour* function in Matlab. In addition, we calculated the overall direction selectivity using the top 75% of the local preferred directions.

For size tuning experiments, we first binned the spiking frequency into 44 ms samples. We then defined the mean spontaneous activity in the bins preceding the stimulus by 500 ms. Peri-stimulus bins which surpassed the mean spontaneous activity were averaged to determine mean spiking responses. For looming experiments, we first calculated the spike histogram using sliding windows with width of 20 ms. We then calculated the maximum response for each neuron, and the time relative to maximum looming stimulus size. To calculate looming selectivity we used the following equation: (Max Response to Looming − Max Response to Control)/(Max Response to Looming + Max Response to Control), where *Control* refers to the luminance matched control (Fig. 3d) or the appearance control (Fig. 3e). For full-screen sinusoidal gratings, and starfield stimuli, we quantified the mean spike frequency for the entire stimulus duration, after removing the first 100 ms of the response, to avoid any initial onset transients (Nordström and O’Carroll 2009b; Nordström et al. 2011).

With the exception of receptive fields, a minimum of three technical replicates were performed for each experiment in each neuron. The mean of these technical replicates constitutes a single biological replicate, and all reported N values represent biological replicates. No data were excluded before calculating the means. Statistical analysis was performed in GraphPad Prism (version 7.0c, GraphPad Software Inc, USA), after ensuring that the data were normally distributed. *p* values below 0.05 were used to refute the null hypothesis.

## Results

**Fig. 1:**
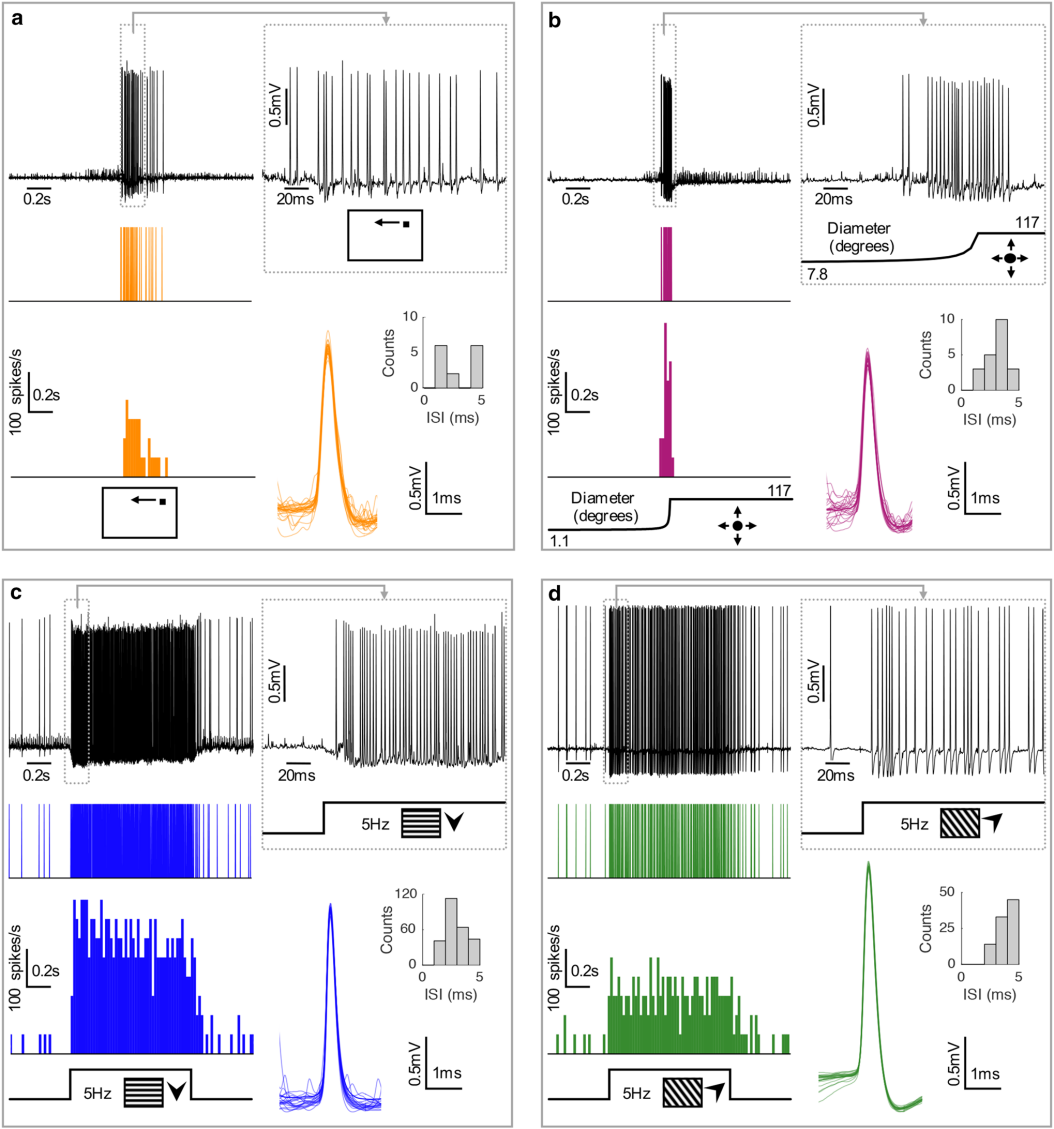
Raw responses of hoverfly descending neurons. **a** Extracellular recording of a target-selective descending neuron (TSDN) in a male hoverfly in response to small target motion in its preferred direction. Top left shows an example raw data trace, with the resulting spike train below, and at the bottom the average spike histogram (in 20 ms bins). Top right shows a magnification of the raw data at the start of the response to target motion. Bottom right shows 20 example individual waveforms, as well as the interspike intervals (ISI), for this recording. **b** A raw data example from a looming sensitive descending neuron in response to a looming stimulus. **c** A raw data example from an optic flow sensitive 1 descending neuron in response to a 5-Hz sinusoidal grating moving in its preferred direction. **d** A raw data example from an optic flow sensitive 2 descending neuron in response to a 5-Hz sinusoidal grating moving in its preferred direction

To characterize visual descending neurons in the hoverfly we performed extracellular recordings (Fig. 1) in the cervical connective while stimulating the animal with visual stimuli. The data were spike sorted using the action potential amplitude and width of individual waveforms (Fig. 1). We found that some descending neurons were selective to the motion of small targets crossing a small part of the visual field (Fig. 1a). Such TSDNs have previously been described in dragonflies, robberflies and hoverflies (Olberg 1981; Gonzalez-Bellido et al. 2013; Nicholas et al. 2018a). Other neurons appeared to be more similar to looming neurons (Fig. 1b), previously described in, e.g. *Drosophila* and locusts (see, e.g. Santer et al. 2008; Peron and Gabbiani 2009; Fotowat et al. 2011; Yakubowski et al. 2016; Zacarias et al. 2018; Ache et al. 2019a, b). Other descending neurons (Fig. 1c, d) shared response properties with optic flow sensitive descending neurons previously described in *Drosophila* and larger flies (see, e.g. Wertz et al. 2008, 2009b; Suver et al. 2016). In the following sections, we will describe how these descending neurons can be identified based on their receptive fields, and how their responses to different visual stimuli vary from each other.

### Descending neurons can be clustered based on their receptive field properties

**Fig. 2:**
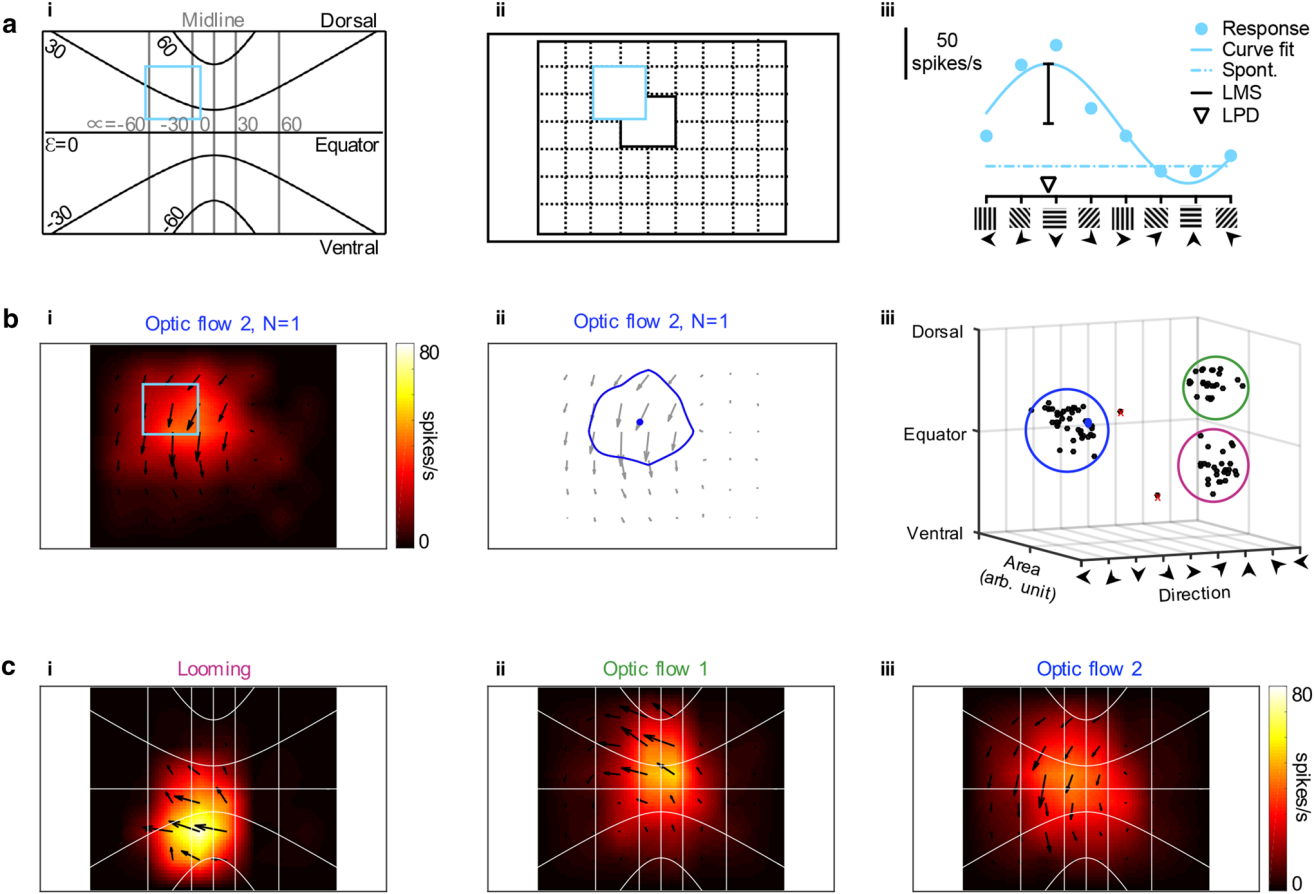
Receptive fields of three clusters of hoverfly descending neurons. **a***i* The hoverfly was placed upside down in front of the visual stimulus screen, at a distance of 6.5 cm. The pictogram illustrates what part of the visual field this corresponds to, where *α* is azimuth, and *ε* elevation in degrees. The light blue box highlights the example used in the following panels. *ii* We divided the central screen into a grid of 48 overlapping squares with 38° sides, in which we displayed sinusoidal gratings moving in eight different directions. *iii* An example response to the eight directions of motion in the part of the screen indicated with a light blue box (panel *i* and *ii*), with a fitted sinusoidal function, the resulting local motion sensitivity (LMS, black line) and local preferred direction (LPD, open arrowhead). **b***i* The resulting receptive field of a single optic flow sensitive 2 neuron, where the arrow highlighted with the blue box corresponds to the panels shown in (**a**). *ii* The center of the receptive field of the same neuron highlighted with a blue circle, and the area of the receptive field with a blue outline. *iii* The center, area and direction preference of each recorded neuron’s receptive field, plotted in 3 dimensions, which highlights three distinct clusters outlined in magenta, blue and green. Two neurons (red crosses) were excluded from further analysis as they did not cluster tightly with the others. The example neuron from previous panels is displayed in blue. **c** Average receptive fields for *i* looming sensitive (*N* = 28), *ii* optic flow sensitive 1 (*N* = 24), and *iii* optic flow sensitive 2 (*N* = 42) neurons

We first mapped receptive fields using small, local sinusoidal gratings (modified from Krapp and Hengstenberg 1997; Straw et al. 2006). For this, a square patch with an average side of 38° was placed in a pseudo-randomly chosen location on the screen (pale blue, Fig. 2a-i) out of 48 overlapping possible positions (Fig. 2a-ii). For each location we also recorded the spontaneous rate preceding stimulation with the sinusoidal grating, which moved in eight different directions. By fitting a cosine function to the mean response for each direction, we could extract the local preferred direction (arrowhead, LPD, Fig. 2a-iii) and the local motion sensitivity (black vertical bar, LMS, Fig. 2a-iii) in each location (modified from Straw et al. 2006). We displayed this as vectors, with the preferred direction given by the vector angle and the sensitivity by its length (Fig. 2b-i). In addition, we calculated the average spiking frequency for each location, and after subtracting the spontaneous rate, we spatially interpolated this ten times (color coding, Fig. 2b-i).

As previously described (Nicholas et al. 2018a), TSDNs do not respond to sinusoidal gratings, so this technique could not be used for mapping their receptive fields. For all other descending neurons that we recorded from (*N* = 96), we extracted the center of each receptive field (blue circle, Fig. 2b-ii), the receptive field size (blue outline, Fig. 2b-ii), and the average preferred direction (gray arrows, Fig. 2b-ii). When plotted in three dimensions the receptive field data clearly form three main clusters, with no overlap (Fig. 2b-iii). Two neurons did not cluster with the others, and were, therefore, removed from further analysis (red crosses, Fig. 2b-iii). We are referring to the three clusters as looming sensitive (*N* = 28, magenta outline, Fig. 2b-iii), optic flow sensitive 1 (*N* = 24, green outline, Fig. 2b-iii) and optic flow sensitive 2 (*N* = 42, blue outline, Fig. 2b-iii), as justified below. All data from these neurons were included in subsequent analyses.

We next averaged the receptive fields of all neurons in each of the three clusters (Fig. 2b-iii). The neurons in the first of these clusters, ‘Looming sensitive’, have receptive fields with highest sensitivity in the ventral visual field, close to the visual midline, with local preferred direction away from the midline (*N* = 28, Fig. 2c-i). The neurons in the second cluster, ‘Optic flow sensitive 1’, have receptive fields in the dorsal visual field with preferred motion away from the visual midline (*N* = 24, Fig. 2c-ii). The local preferred direction pattern (Fig. 2c-ii) appears to follow the elevation lines, suggesting that it should respond optimally to yaw rotations in the dorsal visual field. The ‘Optic flow sensitive 2’s’ receptive field (*N* = 42, Fig. 2c-iii) responds to downward motion across a large part of the visual field. The local preferred direction pattern (Fig. 2c-iii) suggests that it should respond optimally to roll rotations.

### Looming neurons respond to the rapid growth of looming stimuli

**Fig. 3:**
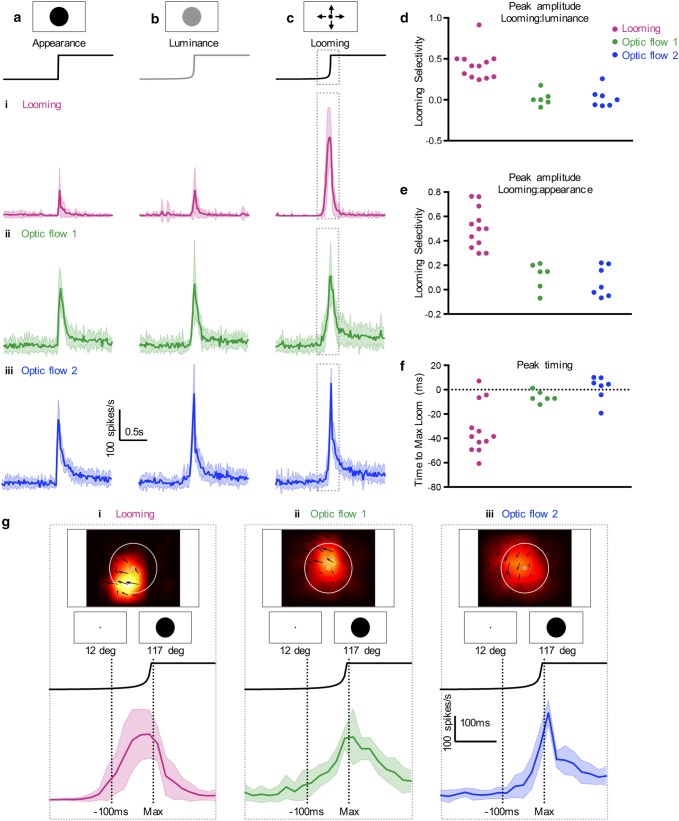
Only looming neurons are tuned to looming stimuli. **a** The response to the appearance of a disc with a diameter of 117°. Row *i* shows data from looming sensitive descending neurons (*N* = 12), rows *ii* and *iii* from optic flow sensitive descending neurons (*N* = 6, and *N* = 7, respectively). The responses are shown as spike histograms of the mean ± std in 20 ms bins. **b** The responses of the same neurons to a luminance matched control. **c** The responses of the same neurons to a looming stimulus with an *l*/|*v*| of 10 ms with a finishing size of 117°. **d** The peak amplitude of the response to the looming stimulus compared to the peak response to the luminance matched control for the neurons displayed in panels (**a**–**c**). **e** The peak response to the looming stimulus compared to the peak response to the appearance control. **f** The peak response relative to the time the looming stimulus reached its maximum size (time = 0, dotted line). **g** A zoomed-in version of the data in panel (**c**) *i*. The two dotted lines indicate the time that the looming stimulus reached maximum size (“Max”) and 100 ms before maximum size (when the diameter of the stimulus was 12°). The pictograms show the relative size of the stimulus at these times, on the screen and compared to the receptive fields of looming sensitive (*i*), optic flow sensitive 1 (*ii*) and optic flow sensitive 2 (*iii*) descending neurons

We found that the descending neurons that we have referred to as looming sensitive (magenta outline, Fig. 2b-iii) respond weakly, or not at all, to the appearance of a large circular disc (magenta data, Fig. 3a-i), with a similarly weak response to a stationary disc whose luminance changes over time (magenta data, Fig. 3b-i). In contrast, the optic flow sensitive neurons respond strongly to both of these stimuli (green and blue data, Fig. 3a, b-ii, iii). When stimulated with a looming stimulus with an *l*/|*v*| of 10 ms both looming and optic flow sensitive neurons respond strongly (Fig. 3c). By comparing the response to the looming stimulus and the luminance-matched control (Fig. 3d) or to the appearance control (Fig. 3e), it is clear that the looming neurons prefer the looming stimulus (magenta data, Fig. 3d, e), similar to looming sensitive neurons described in, e.g. *Drosophila* and locusts (see, e.g. Klapoetke et al. 2017; Dewell and Gabbiani 2018). However, the neurons found in the second and third clusters (blue and green outlines, Fig. 2b-iii) are not responding to the looming as such, but rather to the rapid luminance change associated with the rapidly growing disk (green and blue data, Fig. 3d, e).

We next looked at the timing of the response to the looming stimulus, where we defined zero as the time when the stimulus reached its maximum size (‘Max’, Fig. 3g). We found that 9 out of the 12 looming neurons reached the peak response earlier than the optic flow sensitive neurons and this difference was significant (*p* < 0.001, 2-way ANOVA, Fig. 3f, g). In addition, the looming neurons reached their peak response significantly before the stimulus reached its maximum size (*p* = 0.0024, Wilcoxon signed rank test, Fig. 3f, g), similar to looming neurons described in locusts (Fotowat and Gabbiani 2007), crabs (Oliva and Tomsic 2014), and *Drosophila* (de Vries and Clandinin 2012; von Reyn et al. 2014). Indeed, the looming neurons start firing when the looming disc has a diameter of only 12°, and at the time the disc reaches its maximum diameter of 117°, the firing rate has saturated (magenta data, Fig. 3g-i). In contrast, the optic flow sensitive neurons reached their peak response around the time of maximum size (ns, Wilcoxon signed rank test, green and blue data, Fig. 3f, g), consistent with the hypothesis that they respond to luminance changes rather than looming per se. Note that when the looming stimulus reached its maximal size it covered the majority of the receptive fields of all three neurons (Fig. 3g). TSDNs do not respond to looming stimuli, or to any of the controls.

### Both target-selective and looming neurons respond to small targets

**Fig. 4:**
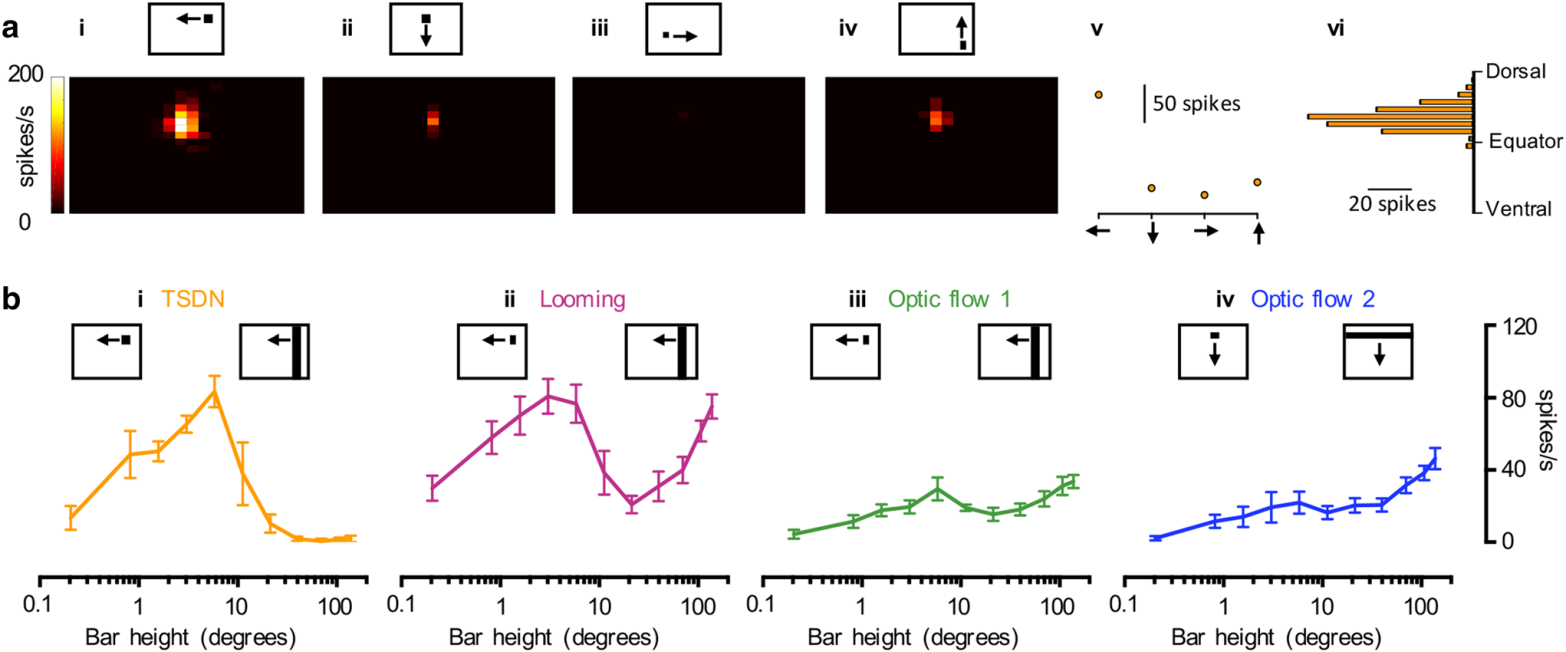
Target-selective descending neurons are uniquely tuned to small stimuli. **a** We scanned the screen with a square target with a 3° side in four different directions (*i*–*iv*) along 20 evenly spaced trajectories. The data show an example response from a single TSDN. *v* The graph shows the average response to each direction of target motion for this example TSDN. *vi* The response to target motion as a function of elevation for this example TSDN. **b***i* Size tuning for TSDNs (*N* = 7) generated by scanning the screen horizontally at 130° per second with a 3° wide target, and varying height, through the center of the receptive field. *ii* Size selectivity for looming sensitive neurons (*N* = 12). *iii* Size selectivity for optic flow sensitive 1 neurons (*N* = 6). *iv* Size selectivity for optic flow sensitive 2 neurons (*N* = 7) generated by scanning the screen vertically with a 3° high target, and varying width. In all panels, the data are displayed as mean ± sem response after subtracting the spontaneous rate

We next investigated the size tuning of the descending neurons. For this purpose we first determined the preferred direction of each neuron, in response to small targets (3° square) scanning the screen vertically and horizontally along 20 evenly spaced trajectories, respectively (modified from Nordström et al. 2006). The example data show a TSDN that responds preferentially to leftward motion (Fig. 4a-i), and less to the other three directions (Fig. 4a-ii–v). In addition, we determined the elevation which gave the strongest response to the small target (Fig. 4a-vi).

We next quantified the size tuning of the descending neurons by scanning a 3°-wide bar through the area of peak sensitivity (Fig. 4a-vi), in each neuron’s preferred direction (Fig. 4a-v). Between scans we varied the height of the bar (the side perpendicular to the direction of travel) in a random order. We found that the size tuning for TSDNs was similar to that previously reported (Nicholas et al. 2018a) with a peak response to bars subtending a few degrees of the visual field and no response to larger bars (*N* = 7, Fig. 4b-i). In contrast, looming sensitive neurons (*N* = 12, Fig. 4b-ii) show a bimodal size selectivity, with one response peak to bars subtending a few degrees of the visual field, similar to the TSDN size tuning (Fig. 4b-i), followed by a dip to larger bars, and then an increased response as the bar was extended to cover the height of the screen. This result does not depend on the analysis window used (data not shown). The optic flow sensitive descending neurons give a stronger response to larger bars with a similar size dependence for optic flow sensitive 1 (*N* = 6, Fig. 4b-iii) and optic flow sensitive 2 (*N* = 7, Fig. 4b-iv). Note that the optic flow sensitive descending neurons did not respond as strongly to bars as the looming neurons did.

### Looming and optic flow sensitive neurons respond to widefield sinusoidal gratings

**Fig. 5:**
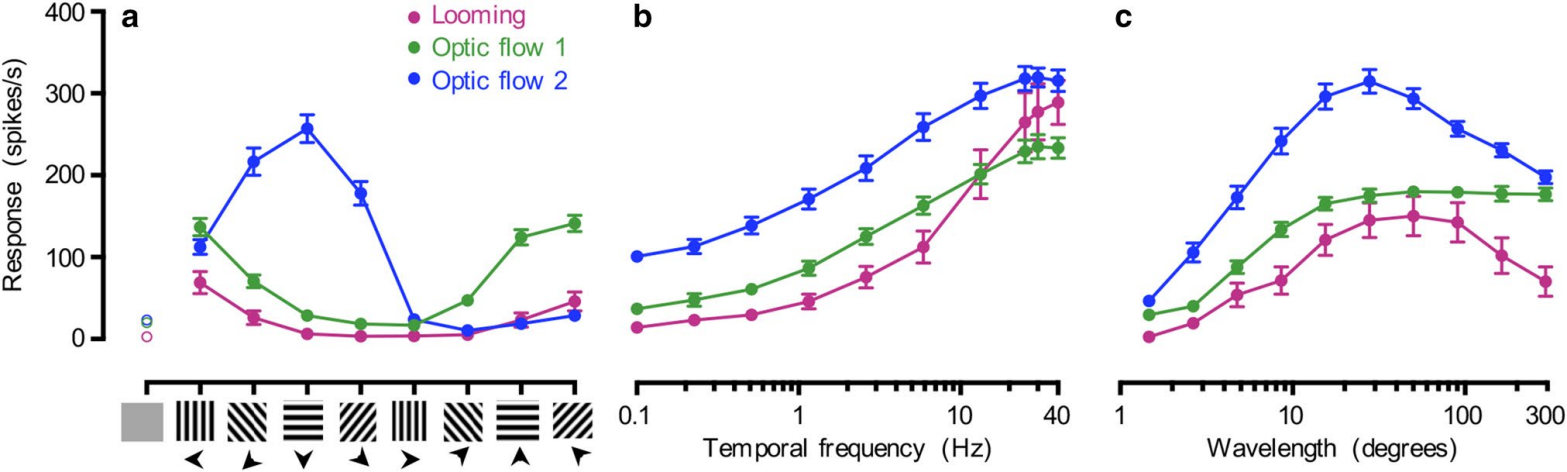
Looming and optic flow sensitive neurons respond to full-screen sinusoidal gratings. **a** Direction tuning in response to a full-screen, full-contrast sinusoidal grating with a wavelength of 7° drifting at 5 Hz, for looming sensitive neurons (magenta, *N* = 16), optic flow sensitive 1 neurons (green, *N *= 16), and optic flow sensitive 2 neurons (blue, *N* = 23). The spontaneous rate is shown with open symbols. **b** Temporal frequency response function to a full-screen, full-contrast sinusoidal grating with a wavelength of 7° drifting in each neuron’s preferred direction (*N* = 13, *N* = 7 and *N* = 11, respectively). **c** Wavelength tuning in response to a full-screen, full-contrast sinusoidal grating drifting at 5 Hz in each neuron’s preferred direction (*N* = 13, *N* = 7 and *N* = 11, respectively). In all panels, the data are displayed as mean ± sem

As described above both optic flow sensitive and looming sensitive descending neurons respond to small sinusoidal gratings (Fig. 2). We next investigated this sensitivity in more detail, using full-screen stimulation. We first determined the direction selectivity by using a sinusoidal grating with a temporal frequency of 5 Hz and a wavelength of 7° and found that looming neurons responded best to leftward motion (magenta data, Fig. 5a), consistent with their receptive fields (Fig. 2c-i). The direction sensitivity of the optic flow sensitive neurons was also consistent with the receptive fields (compare Fig. 5a and Fig. 2c-ii, iii), with a peak response to downward motion for optic flow sensitive 2 (blue data, Fig. 5a), whereas the optic flow sensitive 1 neurons preferred motion at an upwards angle (green data, Fig. 5a). The direction tuning was significantly different between the three neurons (*p* < 0.0001, 2-way ANOVA, Fig. 5a).

When stimulated with gratings with different temporal frequencies, with a wavelength of 7°, the looming neurons did not respond strongly to low temporal frequencies but the response increased vigorously to faster moving sinusoidal gratings (magenta data, Fig. 5b). The temporal frequency tuning of the looming neurons was significantly different from the response of the optic flow sensitive 2 neurons, but not of the optic flow sensitive 1 neurons (2-way ANOVA). The optic flow sensitive 1 neurons’ responses were significantly smaller than the optic flow sensitive type 2 neurons (compare green and blue data, *p* = 0.005, 2-way ANOVA, Fig. 5b).

In response to gratings with different wavelengths, moving at 5 Hz, the looming neurons gave a peak response at a wavelength around 40° (magenta data, Fig. 5c), whereas the optic flow sensitive 2 neurons gave a peak response at a wavelength of 30° (*p* < 0.0001, 2-way ANOVA, blue data, Fig. 5c). The response of the optic flow sensitive 1 neurons appeared to plateau at 20° (green data, Fig. 5c), and the response was significantly different from that of the optic flow sensitive 2 neurons (*p* = 0.0013, 2-way ANOVA). The three clusters of neurons were identified based on their receptive fields (Fig. 2c-iii); thus all respond to full-screen sinusoidal gratings (Fig. 5), whereas TSDNs do not (Nicholas et al. 2018a).

### Looming and optic flow sensitive neurons respond to 3D optic flow

**Fig. 6:**
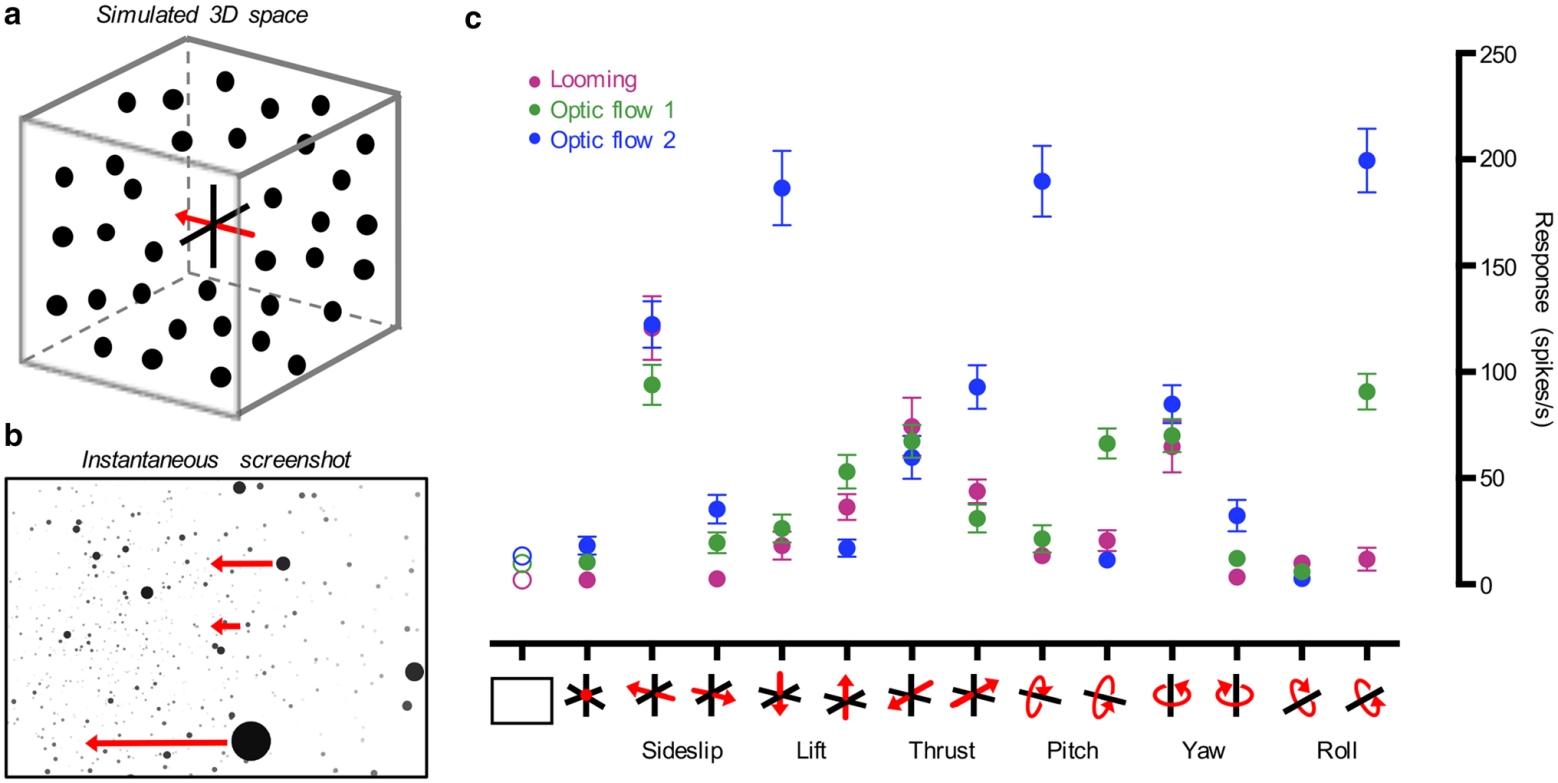
Looming and optic flow sensitive neurons respond to 3D optic flow. **a** We simulated a 3D space, which was shaped like a cube with a 4 m side, and filled with 2 cm spheres at a density of 100 per m^3^, with the hoverfly placed in its center. **b** An example instantaneous screen shot of the resulting pattern on the visual stimulus display, where the size and brightness of the spheres are used to indicate distance from the hoverfly. During translational motion (in this example, sideslip to the left), spheres that are simulated to be close to the hoverfly will move faster across the screen than spheres that are simulated to be further away (as indicated with example red arrows). **c** The response of looming sensitive (magenta, *N* = 14), optic flow sensitive 1 (green, *N* = 9) and optic flow sensitive 2 (blue, *N* = 11) neurons to translations and rotations as indicated with the pictograms. The spontaneous rate is shown with open symbols. In all panels, the data are displayed as mean ± sem

To investigate responses to widefield stimulation in more detail, we developed a perspective distorted, 3-dimensional starfield stimulus. For this purpose, we simulated a 4-m cubic space with the hoverfly placed in its center (Fig. 6a). The cube was filled with 2 cm spheres at a density of 100 per m^3^ (Fig. 6a), in which we simulated different types of optic flow. For example, during leftwards sideslip the entire space slides to the left (Fig. 6a). When this is projected onto the screen in front of the fly, dots that are simulated to be closer to the fly will move faster across the screen (bottom example, Fig. 6b) than dots that are simulated to be further away (central example, Fig. 6b).

We found that the 3-dimensional sideslip (50 cm/s) and yaw (50 °/s) stimuli excited both optic flow sensitive (blue and green, Fig. 6c) and looming sensitive neurons (magenta, Fig. 6c, note that the data points overlap each other). We also found that both looming and optic flow sensitive descending neurons responded well to thrust (Fig. 6c). However, the looming neurons’ responses were larger to a single looming object (magenta, Fig. 3) than to thrust motion (magenta, Fig. 6c). The optic flow sensitive 2 neurons responded strongly to lift and pitch motion (blue, Fig. 6c), whereas the optic flow sensitive 1 neurons responded to pitch in the opposite direction (green, Fig. 6c). Three-dimensional roll motion gave the most clearly differentiated responses, where both optic flow sensitive neurons responded, but looming neurons did not (Fig. 6c). The TSDNs did not respond to any of the optic flow stimuli.

## Discussion

We have described visual motion-sensitive descending neurons in the hoverfly *Eristalis tenax* (Fig. 1). Using the receptive field properties as mapped with small sinusoidal gratings, we identified three distinct clusters of neurons (Fig. 2). The neurons in one of these clusters responded stronger to looming stimuli than to controls (magenta, Fig. 3) and are, therefore, referred to as looming sensitive descending neurons. The neurons in the other two clusters responded with equal strength to both looming stimuli and the luminance-matched or appearance controls (green and blue, Fig. 3). As these neurons had receptive fields (Fig. 2c-ii, iii) similar to previously described neurons in other flies (Wertz et al. 2008, 2009b; Suver et al. 2016), and responded similarly to widefield optic flow (green and blue data, Figs. 5, 6), they are referred to as optic flow sensitive descending neurons. TSDNs are, as previously described (Nicholas et al. 2018a), defined by their selective response to the motion of small targets (Fig. 4).

### Descending optic flow sensitive neurons

Self-generated optic flow is processed by large tangential cells in the lobula plate, the LPTCs. Two types of LPTCs have been especially well studied, namely those belonging to the horizontal system (HS) and the vertical system (VS). *Drosophila* HS cells synapse with DNHS1 (Suver et al. 2016), which is also called DNp15 (Namiki et al. 2018). The receptive field of the optic flow sensitive 1 neuron (Fig. 2c-ii) suggests that it receives input mainly from the dorsal HS neurons in the hoverfly lobula plate (Nordström et al. 2008). Furthermore, the optic flow sensitive 1 neuron responds strongly to yaw and roll, as well as to horizontal motion of a sinusoidal grating (green data, Figs. 5, 6c), similar to DNHS1 (Suver et al. 2016). It is thus possible that the optic flow sensitive 1 descending neuron (green data, Figs. 1, 2, 3, 4, 5, 6) is the hoverfly homologue of *Drosophila* DNHS1.

*Drosophila* and blowfly VS cells synapse with the DNOVS2 neuron (Wertz et al. 2008, 2009b; Suver et al. 2016), which is also called DNp22 (Namiki et al. 2018). The blowfly DNOVS2 neuron gives stronger responses to roll than to lift (Wertz et al. 2009a, b). In *Drosophila*, DNOVS2 responds strongly to both roll and pitch and gives smaller responses to thrust and yaw (Suver et al. 2016). The optic flow sensitive 2 neuron described here responded to sideslip, thrust and yaw, but even stronger to lift, pitch and roll (blue data, Fig. 6c). Together with its receptive field (Fig. 2c-iii), these results suggest that it might be the hoverfly homologue of DNOVS2. Another dipteran descending neuron receiving input from VS cells is DNOVS1 (Wertz et al. 2008, 2009b; Suver et al. 2016), which is also called DNp20 in *Drosophila* (Namiki et al. 2018). As DNOVS1 produces graded responses and not action potentials (Wertz et al. 2009b; Suver et al. 2016), it is unlikely to correspond to either of the optic flow sensitive neurons described here (green and blue data, Figs. 1, 2, 3, 4, 5, 6).

The optic flow sensitive descending neurons spatially pool input from LPTCs (Wertz et al. 2008, 2009b; Suver et al. 2016) and thereby have larger receptive fields. In addition, they inherit some of their response properties, such as direction selectivity (Fig. 5a). However, other response properties are clearly different from their presynaptic LPTCs. For example, the descending neurons respond to higher wavelengths (Fig. 5c) than the presynaptic LPTCs, where male *Eristalis* HS cells peak at 5°–10° (Straw et al. 2006). *Eristalis* LPTCs do respond to much larger wavelengths, but the response is much smaller than to 5°–10° wavelengths (Straw et al. 2006). In addition, the descending neurons respond to higher temporal frequencies (Fig. 5b), than their presynaptic LPTCs, where male *Eristalis* HS cells peak at 20 Hz, and the response rapidly declines at higher frequencies (Straw et al. 2006). Importantly, hoverfly LPTCs are unique in their ability to simultaneously respond to high temporal frequencies and long wavelengths, which has been argued to be an adaptation that allows for the alternate demands of hovering at very low velocities and pursuing conspecifics at high-speed (O’Carroll et al. 1996). The shift to even higher temporal frequencies (Fig. 5b) and wavelengths (Fig. 5c) in the descending neurons suggests that additional processing may be in place beyond the LPTCs.

The output synapses of most LPTCs are found in the posterior slope of the brain (Namiki et al. 2018). Following retrograde tracing of descending neurons, the posterior slope is the most densely labelled of all brain regions (Hsu and Bhandawat 2016). From the posterior slope, descending neurons, including DNOVS2 and DNHS1, project to the thoracic ganglia, where locomotion of the neck, wing and halteres is controlled (Suver et al. 2016; Namiki et al. 2018). In addition, there are many pre-motor interneurons and sensory neurons from mechanoreceptors in these areas (Namiki et al. 2018; Venkatasubramanian and Mann 2019). Biocytin labelling showed that DNOVS1 and DNOVS2 are coupled to frontal nerve neck motor neurons (Strausfeld and Bassemir 1985; Gronenberg et al. 1995; Suver et al. 2016). Since the optic flow sensitive neurons that we recorded from (green and blue data, Figs. 1, 2, 3, 4, 5, 6) are likely to be the hoverfly homologues of DNHS1 and DNOVS2, a direct influence on the head and wing is, therefore, likely. Indeed, optogenetic activation of HS cells, pre-synaptic to DNHS1 (Suver et al. 2016), leads to yaw motion of both the head and the body (Haikala et al. 2013).

### Looming neurons

The neural underpinnings of a rapid escape response to a looming stimulus have been particularly well investigated in locusts, crabs and *Drosophila* (see, e.g. Card 2012; Tomsic 2016). In locusts, the looming sensitive lobula giant movement detector (LGMD) connects with the descending contralateral movement detector (DCMD, Rowell et al. 1977). LGMD spikes are conveyed one-to-one to the DCMD (Rind and Simmons 1999), which in turn prepares hind-leg flexion for an escape jump (Santer et al. 2008).

In *Drosophila*, looming sensitive neurons have been found at multiple levels of the visual processing pathway. For example, LPLC2, which are looming sensitive visual projection neurons (Klapoetke et al. 2017), as well as Foma-1 neurons (de Vries and Clandinin 2012), are found in the lobula complex, whereas LC4 neurons are found in the optic glomeruli (von Reyn et al. 2014). LC4 neurons (von Reyn et al. 2014) and LPLC2 neurons provide direct input to the giant fiber (Klapoetke et al. 2017; Ache et al. 2019b), also referred to as DNp01 (Namiki et al. 2018). However, there are several other looming sensitive descending neurons in *Drosophila,* whose responses are modulated by behavioral state. For example, the activation of looming sensitive DNp07 and DNp10 neurons leads to a take-off response if the fly is standing, but a landing response if the fly is in flight (Ache et al. 2019a). Similarly, flies in a constrained area either freeze or run away from a looming stimulus depending on their walking speed at the start of the loom, with the freezing behavior supported by DNp09 (Zacarias et al. 2018). This highlights the fact that the descending neurons do not just provide a bottleneck of information between the roughly 100,000 neurons in the *Drosophila* brain (Zheng et al. 2018) and the 1100 descending neurons (Hsu and Bhandawat 2016), but they must also incorporate additional higher order processing, including decision-making and learning. For example, the *Drosophila* loom sensitive AX neuron’s activity is correlated with visually elicited, as well as with spontaneous, saccadic turns (Schnell et al. 2017).

Foma-1 neurons respond to global motion as well as to looming stimuli (de Vries and Clandinin 2012), whereas LPLC2 do not (Klapoetke et al. 2017). As the looming sensitive neurons responded strongly to both sinusoidal gratings (magenta data, Fig. 5) and to 3D optic flow (magenta data, Fig. 6), they share many properties with optic flow sensitive neurons. In the future it would be interesting to investigate the interactions between background optic flow and a looming stimulus, similar to what has been done in locusts (Yakubowski et al. 2016). Notable, however, is the finding that the looming neurons responded much stronger to discrete objects than optic flow neurons did (Fig. 4) and that they responded stronger to a single looming object (magenta data, Fig. 3c) than to thrust motion (magenta data, Fig. 6), even though both contain approaching cues. In our 3D optic flow (Fig. 6) we displayed many smaller objects that were simulated to be further away than 6 cm, which might explain this discrepancy.

The looming sensitive neurons that we recorded from had receptive fields close to the visual midline, with directional sensitivity to motion away from the midline (Fig. 2c-i). *Drosophila* LPLC2 neurons, which are pre-synaptic to the giant fiber, have small receptive fields that tile the visual field (Klapoetke et al. 2017). Foma-1 neurons have dorsal, directional receptive fields (de Vries and Clandinin 2012), suggesting that hoverfly homologues of either of these could provide input to the neurons that we recorded from.

### Small target sensitivity

TSDNs have been described in dragonflies as well as in hoverflies and robberflies (Olberg 1981, 1986; Gonzalez-Bellido et al. 2013; Nicholas et al. 2018a). Target responses in themselves are not enough to identify a neuron as a TSDN, as many looming sensitive neurons also respond to small targets, including ours (Fig. 4b-ii), the locust LGMD/DCMD neurons (Rowell et al. 1977), crab lobula giant neurons (Medan et al. 2007), and looming sensitive locust central complex neurons (Rosner and Homberg 2013).

It has been speculated that lobula STMDs are directly presynaptic to TSDNs (Nordström and O’Carroll 2009a), even if direct evidence is lacking. This hypothesis is supported by the receptive fields of TSDNs which are located in the dorso-frontal visual field (Fig. 4a), where small-field lobula small target motion detectors (STMDs) have their receptive fields (Barnett et al. 2007). Furthermore, STMDs give peak responses to 1°–3° high bars, and TSDNs to 3°–6° high bars, i.e. in a similar range (Fig. 4b-i, and see also Barnett et al. 2007; Nicholas et al. 2018a). Importantly, however, the input dendrites of many descending neurons overlap with the optic glomeruli (Hsu and Bhandawat 2016; Namiki et al. 2018), which encode distinct visual features, including small objects (von Reyn et al. 2014; Aptekar et al. 2015; Wu et al. 2016; Klapoetke et al. 2017), suggesting that TSDN input could come from these rather than directly from STMDs.

## Conclusion

Recent work has suggested that population responses of descending neurons are probably more likely to drive behavioral output, than the action of individual ‘command neurons’ (Gonzalez-Bellido et al. 2013; Namiki et al. 2018). The response properties of the neurons that we have described suggest that several of them would typically fire during any given stimulus condition. Depending on the motor neurons they provide input to, this would allow a large repertoire of behaviors.

## Abbreviations

3D: 3-dimensional
DCMD: Descending contralateral movement detector
DNHS1: Descending neuron of the horizontal system 1
DNOVS: Descending neuron of the ocellar and vertical system
HS: Horizontal system
ISI: Interspike interval
*l*/|*v*|: Object half-size divided by approach speed
LGMD: Lobula giant movement detector
LMS: Local motion sensitivity
LPD: Local preferred direction
LPTC: Lobula plate tangential cell
STMD: Small target motion detector
TSDN: Target-selective descending neuron
VS: Vertical system
